# Exploring rose absolute and phenylethyl alcohol as novel quorum sensing inhibitors in *Pseudomonas aeruginosa* and* Chromobacterium violaceum*

**DOI:** 10.1038/s41598-024-66888-z

**Published:** 2024-07-08

**Authors:** Halime Çevikbaş, Seyhan Ulusoy, Neslihan Kaya Kinaytürk

**Affiliations:** 1 Faculty of Engineering and Natural Sciences, Biology Department, Süleyman Demirel University, Isparta, 32260 Turkey; 2 Faculty of Arts and Science, Nanoscience and Nanotechnology Department, Mehmet Akif Ersoy University, Burdur, 15100 Turkey

**Keywords:** Phenylethyl alcohol, Rose absolute, *Pseudomonas aeruginosa*, Biofilm, *Chromobacterium violaceum*, Molecular docking, Bacteria, Biofilms

## Abstract

Inter-cellular signaling, referred to as quorum sensing (QS), regulates the production of virulence factors in numerous gram-negative bacteria, such as the human pathogens *Pseudomonas aeruginosa* and *Chromobacterium violaceum*. QS inhibition may provide an opportunity for the treatment of bacterial infections. This represents the initial study to examine the antibiofilm and antivirulence capabilities of rose absolute and its primary component, phenylethyl alcohol. QS inhibition was assessed by examining extracellular exopolysaccharide synthesis, biofilm development, and swarming motility in *P. aeruginosa* PAO1, along with violacein production in *C. violaceum* ATCC 12472. Molecular docking analysis was conducted to explore the mechanism by which PEA inhibits QS. Our results indicate that rose absolute and PEA caused decrease in EPS production (60.5–33.5%), swarming motility (94.7–64.5%), and biofilm formation (98.53–55.5%) in the human pathogen *P. aeruginosa* PAO1. Violacein production decreased by 98.1% and 62.5% with an absolute (0.5 v/v %) and PEA (2 mM). Moreover, the molecular docking analysis revealed a promising competitive interaction between PEA and AHLs. Consequently, this study offers valuable insights into the potential of rose absolute and PEA as inhibitors of QS in *P. aeruginosa* and *C. violaceum*.

## Introduction

*Pseudomonas aeruginosa*, an opportunistic pathogen, causes nosocomial infections and significantly impacts individuals with compromised immune systems, such as cystic fibrosis patients^1,2^. This bacterium synthesizes various virulence factors, including LasB elastase, pyoverdin, and pyocyanin, and regulates these through quorum sensing (QS)^3–8^. QS also controls motility, biofilm formation, and antibiotic resistance, making *P. aeruginosa* particularly resilient against treatment^9^. Similarly, *Chromobacterium violaceum*, a saprophytic bacterium, is known to cause infections in humans and animals^10,11^. It produces violacein, a QS-regulated compound, useful in studying anti-quorum sensing effects of different substances^12^.

Given the rise in antibiotic-resistant strains, nonantibiotic therapies are urgently needed. QS inhibitors, which reduce bacterial pathogenicity without affecting growth, offer a promising strategy^13–19^. Recent studies have identified natural antibiofilm agents effective against *P. aeruginosa* and *C. violaceum*^20–27^.

*Rosa damascena* Mill., or damask rose, is widely used in herbal medicine. Its extracts, such as rose essential oil and rose absolute, have shown various biological effects, including antibacterial and antioxidant properties^25,26,28–32^. Phenylethyl alcohol (PEA), a major component of rose absolute, is also present in many plants and is recognized as safe for use in food and pharmaceuticals^33–37^. Despite its known antimicrobial properties, the antibiofilm potential of PEA is not well-explored^38–40^.

This study aims to evaluate the anti-quorum sensing properties of rose absolute and PEA against *P. aeruginosa* and *C. violaceum*. We will assess their effects on EPS production, swarming motility, antibiofilm activity, and violacein production, complemented by molecular docking studies to elucidate the mechanisms of action.

## Materials and methods

### Rose absolute

Rose absolute (RA) was obtained from Sebat Ltd., Isparta. Phenylethyl alcohol (PEA) (W285803) was sourced from Sigma–Aldrich. Dimethyl sulfoxide (DMSO) was purchased from Merck, and Luria Bertani Broth (LB) was used for bacterial culture.

### Bacterial strains

*Pseudomonas aeruginosa* (PAO1) and *Chromobacterium violaceum* ATCC 12472 were acquired from the Department of Biology, Süleyman Demirel University, Isparta, Türkiye. Bacterial strains were cultured on LB broth or LB agar plates at 37 °C for 24 h and stored at 4 °C for further use.

### GC–MS analysis

The principal compounds in rose absolute were identified using gas chromatography-mass spectrometry (GC–MS). An Agilent 7890B/5977B GC/MS system with a ZB-WAX plus column (60 m × 250 µm, 250 nm; Phenomenex) was used. Helium (≥ 99.999%) was the carrier gas at a constant flow rate of 1.2 mL/min. Samples (1.0 µL) were injected in split mode (2:1, 250 °C). The ion source temperature was set to 150 °C with electron impact ionization at 70 eV.

### Antibacterial assay

The antibacterial properties of rose absolute (dilutions: 1:5, 1:10, 1:20, 1:100 v/v) and PEA (16, 8, 4, 2 mM) were assessed using well-diffusion assays. *P. aeruginosa* and *C. violaceum* were cultured in LB broth at 37 °C for 24 h. Cultures were adjusted to the McFarland no. 0.5 standard. LB agar (0.3% w/v) was inoculated with 100 µL of bacterial culture and poured onto pre-warmed LB agar plates. Wells were created using a sterile cork borer, and 50 µL of rose absolute or PEA was added. Plates were incubated at 37 °C for 24–48 h, and zones of inhibition (mm) were measured. DMSO served as the negative control, and gentamicin (10 µg/ml) was the positive control.

### MIC assay

The minimum inhibitory concentrations (MICs) of rose absolute and PEA were determined. Twofold serial dilutions (rose absolute: 4% to 0.03% v/v; PEA: 16 to 0.125 mM) were prepared in LB broth^41^. Tubes were inoculated and incubated at 37 °C for 24–48 h. The MIC was defined as the lowest concentration with no visible bacterial growth. Experiments were replicated three times.

### Biofilm formation

Biofilm formation was quantified following established protocols^42^. *P. aeruginosa* (OD_600_ = 1.0) was combined with various concentrations of rose absolute (0%, 0.5%, 0.25%, 0.125%, 0.0625% v/v) or PEA (2, 1, 0.5, 0.25 mM) in microfuge tubes and incubated at 37 °C for 2 days. Tubes were rinsed with sterile distilled water, air-dried, stained with 0.1% crystal violet for 30 min, washed, and destained with 95% ethanol. Absorbance at 570 nm was measured to quantify biofilm formation. Biofilm inhibition was calculated using the formula:

$$ {\text{Biofilm}}\;{\text{inhibition}}\left( \% \right) \, = \left[ {\left( {{\text{Control}}\;{\text{OD}}570\;{\text{nm}} - {\text{treated}}\;{\text{OD}}570\;{\text{nm}}} \right)/{\text{Control}}\;{\text{OD}}570\;{\text{nm}}} \right]{ } \times { }100 $$

### Swarming motility assay

Swarming motility was assessed using swarm plates (1% tryptone, 0.5% yeast extract, 0.5% NaCl, 0.5% agar). A 5 µL bacterial culture (OD_600_ = 1) was inoculated onto swarm plates containing varying concentrations of rose absolute (0%, 0.5%, 0.25%, 0.125%, 0.0625% v/v) or PEA (2, 1, 0.5, 0.25 mM). Plates were incubated at 37 °C, and swarming zones were measured.

### Colony forming unit (CFU) assay

CFU assays were conducted to determine the effect of rose absolute and PEA on bacterial cell counts. *P. aeruginosa* and *C. violaceum* were cultured with rose absolute (1%, 0.5%, 0.25%, 0.125%, 0.0625% v/v) or PEA (2, 1, 0.5, 0.25 mM) for 24 h at 37 °C. Cultures were serially diluted to 10^–12^ and plated on LB agar^43^.

### The EPS production

EPS production was measured using the Sulfuric Acid–UV method^44^. Bacterial cultures were treated with 3 mL of sulfuric acid, vortexed, chilled on ice, and the optical density was measured at 315 nm.

### Inhibition of violacein production

Violacein production by *C. violaceum* ATCC 12472 was assessed using flask-incubation assays^45^. Cultures were supplemented with rose absolute or PEA (0%, 0.5%, 0.25%, 0.125%, 0.0625% v/v and 2, 1, 0.5, 0.25 mM, respectively) and incubated at 37 °C. Violacein was extracted, dissolved in DMSO, and quantified at 585 nm.

### Computational details and molecular docking analysis

High-resolution crystal structures of PDB IDs 2UVO, 6D6L, 6D6A, 6D6O and 6D6P, 4EY15, 4EY17, 3QP2, and 3QP5 proteins were acquired from the protein database^46^. Molecular docking simulations were carried out utilizing Autodock Vina^47^ and receptor-ligand interactions were imagined with Discovery Studio Visualizer^48^.

### Statistical analyses

Data were analyzed using Origin version 2019, with one-way ANOVA and Post-Hoc Bonferroni's test. Results are presented as mean ± standard deviation (SD) of triplicate values, with error bars representing SD. Statistical significance was set at *P* < 0.05.

## Results and discussion

The components and their identified compounds in rose absolute are detailed in Table 1. Through GC–MS analysis, we identified and quantified 13 constituents, representing 96.58% of the total sample. Notably, phenylethyl alcohol (PEA) was the most abundant compound, comprising 63.43% of the composition, followed by citronellol (12.99%), geraniol (6.82%), nonadecane (3.68%), and nerol (3.03%).

**Table 1:** Chemical composition of rose absolute analyzed by GC–MS.

Compound	%	Molecular formula
Ethanol	0.72	C_2_H_5_OH
Heptadecane	0.87	C_17_H_36_
Citronellol	12.99	C_10_H_20_O
Nerol	3.03	C_10_H_18_O
Geraniol	6.82	C_10_H_18_O
Benzyl alcohol	1.05	C_7_H_8_O
Nonadecane	3.68	C_19_H_40_
Nonadecene	1.26	C_19_H_38_
PEA	63.43	C_8_H_10_O
Eicosane	0.17	C_20_H_42_
Methyl eugenol	0.64	C_11_H_14_O_2_
Heneicosane	0.48	C_21_H_44_
Eugenol	1.44	C_10_H_12_O_2_
Totaly	96.58	

### Antibacterial effects

In this study, we examined the antibacterial properties of rose absolute and PEA against *Pseudomonas aeruginosa* (PAO1) and *Chromobacterium violaceum* ATCC 12472.

**Figure 1:**
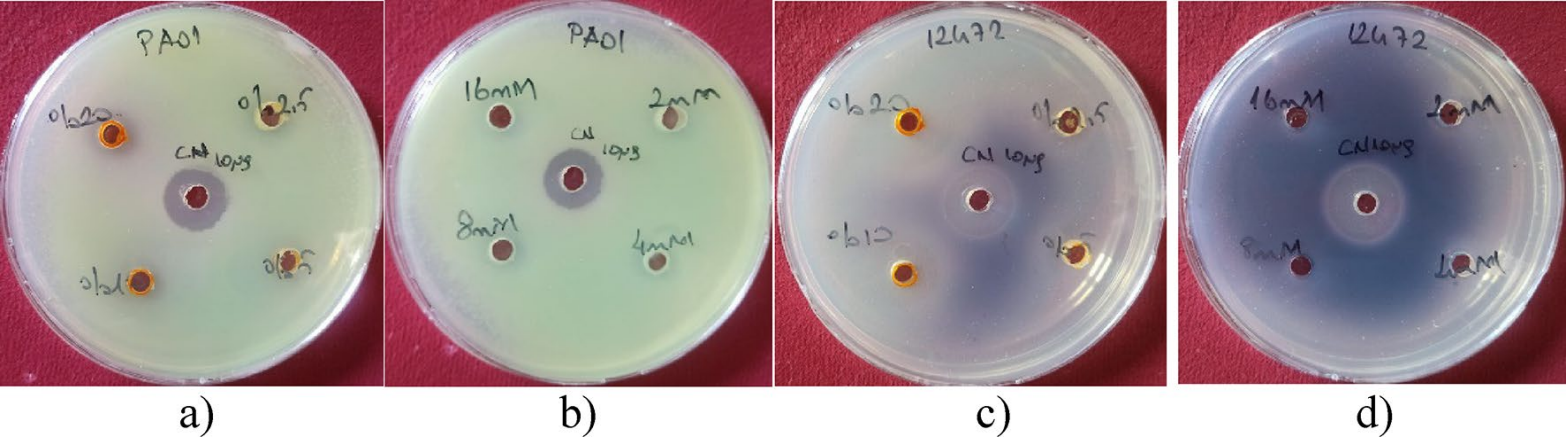
Inhibition zones of rose absolute (2.5–20% (v/v)) and PEA (16 (2 mM), 8 (1 mM), 4(0.5 mM), 1(0.25 mM) against *P. aeruginosa* [(**a**) for rose absolute, (**b**) for PEA] and *C. violaceum* [(**c**) for rose absolute, (**d**) for PEA]. CN represents the positive control gentamicin (10 µg/mL).

Previous studies have documented the antibacterial efficacy of rose absolute and PEA against various Gram-negative and Gram-positive bacteria^30,39,49^. In this study, we examined the antibacterial characteristics of rose absolute and PEA against *P. aeruginosa* and *C. violaceum*. Rose absolute exhibited significant antibacterial potential against *C. violaceum* (Table 2 and Fig. 1). The maximum inhibition zone for rose absolute was 20 ± 0.7 mm, with MIC values of 1.0% (v/v) against *C. violaceum.* PEA and DMSO (control) did not exhibit inhibitory effects on either bacterial strain.

**Table 2:** Inhibition zone diameters (mm) of rose absolute (20 v/v, 10 v/v, 5 v/v, 2.5 v/v %) and PEA (2, 1, 0.5, and 0.25 mM), and the MIC values against *Pseudomonas aeruginosa* PAO1 and *Chromobacterium violaceum*.

	*P. aeruginosa* PAO1					*C. violaceum* ATCC 12472				
Rose absolute (v/v %)	20	10	5	2.5	MIC (v/v %)	20	10	5	2.5	MIC (v/v %)
Inhibition zone (mm)	*	*	*	*	2	20 ± 0.7	15	9.5 ± 0.7	9	1
PEA (mM)	2	1	0.5	0.25		2	1	0.5	0.25	
Inhibition zone (mm)	*	*	*	*	> 16 mM	*	*	*	*	> 16 mM
Gentamicin10µg	15 ± 0.3					18 ± 0.5				

(*): not detected DMSO was used as a negative control.

The colony-forming unit (CFU) assay indicated no significant decrease in cell count variation between control and treated cells with rose absolute (0.5%, 0.25%, 0.125%, 0.625% v/v) or PEA (2, 1, 0.5, 0.25 mM) against *P. aeruginosa* and *C. violaceum*.

### Influence of rose absolute and PEA on biofilm formation

**Figure 2:**
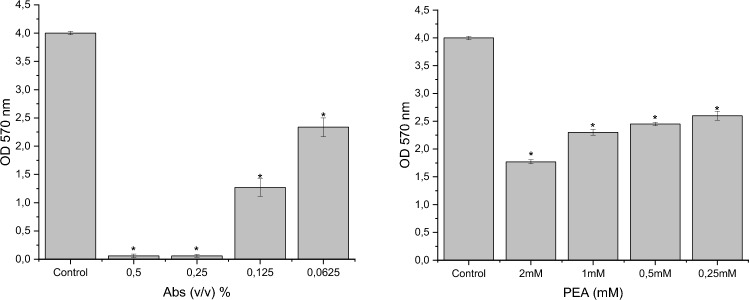
Effect of rose absolute (0.5 v/v, 0.25 v/v, 0.125 v/v, 0.625 v/v %) and PEA (2, 1, 0.5, and 0.25 mM) on the biofilm formation activity of *P. aeruginosa* PAO1. *p < 0.05: Significance compared with control.

Biofilm development and resistance to antimicrobial agents contribute to chronic infections^50^. *P. aeruginosa* regulates biofilm formation through quorum sensing (QS) systems^51,52^. We investigated the impact of rose absolute and PEA on biofilm formation by *P. aeruginosa*. Rose absolute at concentrations of 0.5%, 0.25%, 0.125%, and 0.625% (v/v) reduced biofilm formation by 98.53%, 98.51%, 68.3%, and 41.6%, respectively. Similarly, PEA at concentrations of 2, 1, 0.5, and 0.25 mM resulted in reductions of 55.5%, 42.5%, 38.8%, and 35%, respectively (Fig. 2). This is the first study to demonstrate that subinhibitory concentrations of rose absolute and PEA inhibit biofilm formation in *P. aeruginosa* PAO1.

### Inhibition of *P. aeruginosa* swarming motility by rose absolute and PEA

Bacterial motility plays a crucial role in biofilm formation, and the swarming ability of *P. aeruginosa* depends on type IV pili and flagella^53^. Compromised swarming ability correlates with reduced biofilm formation^54^.

**Figure 3:**
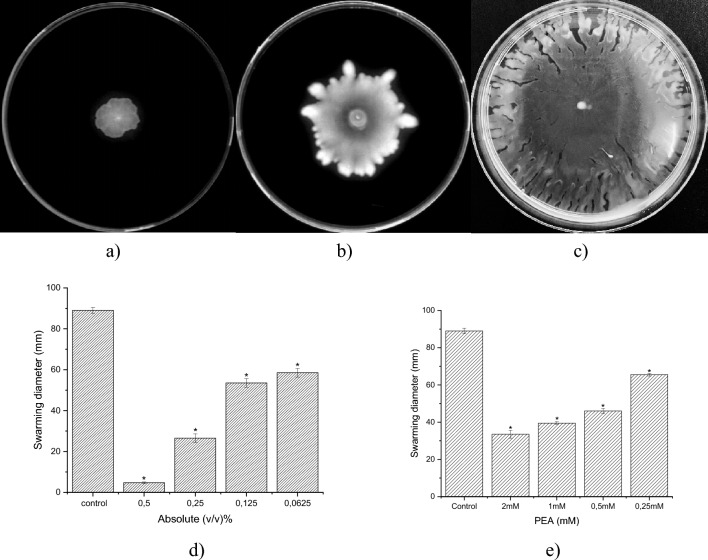
Swarming of *P. aeruginosa* PA01 on LB swarming agar plates with 0.5%(v/v) of rose absolute (**a**), with 2 mM PEA (**b**) and without rose absolute and PEA (**c**). Quantitative estimations of swarming motility inhibition by rose absolute (**d**), by PEA (**e**). *p < 0.05: Significance compared with control.

Citronellol, geraniol, and nerol have been reported to inhibit swarming motility in *P. aeruginosa*^55^*.* We found that rose absolute (0.5% v/v) and PEA (2 mM) inhibited swarming motility by 94.7% and 62.4%, respectively (Fig. 3). This study is the first to demonstrate that rose absolute and PEA inhibit swarming motility in *P. aeruginosa* PAO1.

### EPS production

**Figure 4:**
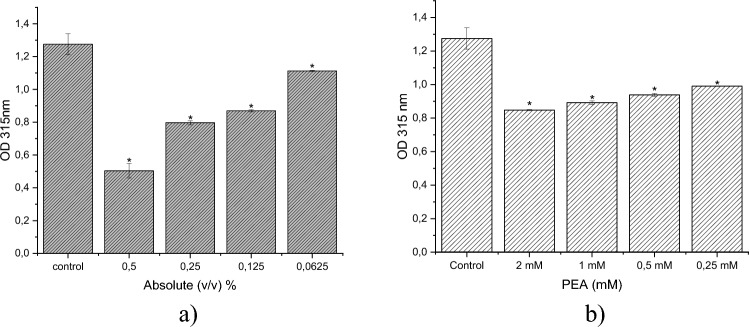
Effect of rose absolute (0.5 v/v, 0.25 v/v, 0.125 v/v, 0.625 v/v %) and PEA (2, 1, 0.5, and 0.25 mM) on exopolysaccharide (EPS) production by *P. aeruginosa* PAO1. *p < 0.05: Significance compared with control.

Extracellular polymeric substance (EPS) is crucial for biofilm structure, microcolony formation, and resistance to antimicrobial agents^56–58^. We demonstrated that sub-inhibitory concentrations of rose absolute and PEA reduced EPS production in *P. aeruginosa* after 24 h of treatment. Using the sulfuric acid method, rose absolute and PEA significantly decreased EPS production by 60.5% and 33.5%, respectively (*p* > 0.05) (Fig. 4). Similarly, Musthafa et al. (2012) reported a decrease in EPS production by approximately 54% in *P. aeruginosa* PAO1 following treatment with phenylacetic acid^59^.

### Violacein inhibition assay

**Figure 5:**
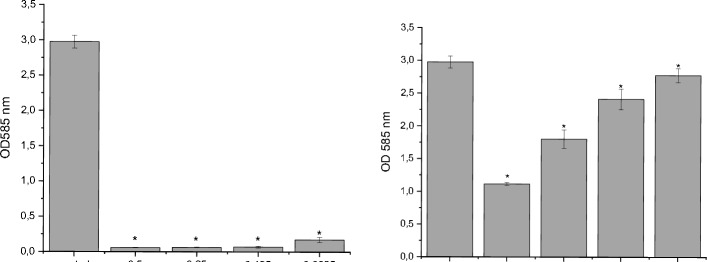
Quantitative assessment of violacein inhibition in *Chromobacterium violaceum* ATCC 12472 by rose absolute (0%, 0.5%, 0.25%, 0.125%, and 0.0625% (v/v)) and PEA (2, 1, 0.5, 0.25 mM). *p < 0.05: Significance compared with control.

*Chromobacterium violaceum* synthesizes violacein through a QS system controlled by the CviR-mediated QS mechanism^60^. An ideal QS inhibitor should not disrupt normal bacterial growth to prevent bacterial resistance^61,62^. We evaluated the anti-QS activity of rose absolute and PEA on violacein production in *C. violaceum* ATCC 12472. At sub-MIC concentrations, neither rose absolute nor PEA inhibited bacterial growth. Rose absolute and PEA inhibited violacein production by 98.1% to 94.2% and 62.5% to 6.7%, respectively, in a concentration-dependent manner (Fig. 5). Previous studies have shown that *Capparis spinosa* extract (2 mg/mL) inhibited violacein production by up to 88%^63^, that Psidium guajava and curcumin inhibited violacein production by *C. violaceum*^64,65^. Our results indicate that rose absolute (0.5% v/v) and PEA (2 mM) significantly decreased violacein levels by 98.1% and 62.5%, respectively (Fig. 5).

### Molecular docking

Molecular docking analysis, a simulation technique, elucidates interactions between receptors (proteins, nucleic acids) and ligands (compounds)^66^. This computational approach generates scores that reflect potential energy changes upon the interaction between a protein and a ligand. Steric interactions, metal ions, and hydrogen bonds collectively influence the resulting score. As shown in Table 3, lower scores (more negative) signify stronger binding affinities^67^.

**Table 3:** The findings from the molecular binding analysis of various LasR and RhlR structures from *P. aeruginosa* PAO1, along with CviR and CviR' proteins from *C. violaceum* ATCC 12472, with PEA, furanone C30, OdDHL, BHL, and AHL are presented.

Protein-structure	Molecule	Binding score kcal/mol	Binding residue
LasR-6D6L	PEA	− 7.2	SER129 THR115 LEU110 ALA105 PHE102 PHE101 TRP88 THR75 ASP73 TYR64 TRP60 TYR56
	OdDHL	− 7.8	SER129 ALA127 THR115 LEU110 ALA105 PHE102 PHE101 TRP88 THR75 ASP73 ALA70 ASP65 TYR64 ARG61 TRP60 TYR56 ILE52 TYR47 LEU36
	Furanone C30	− 6.6	SER129 THR115 LEU110 ALA105 PHE101 PHE102 ILE92 TRP88 THR75 ASP73 TRP60 TYR56
LasR-6D6A	PEA	− 6.7	SER129 ALA127 LEU110 THR115 ALA105 PHE101 TYR93 TRP88 VAL76 THR75 ASP73 TYR64 TRP60 TYR56
	OdDHL	− 8.0	SER129 ALA127 THR115 LEU110 ALA105 PHE101 TYR93 TRP88 VAL76 THR75 ASP73 ALA70 TYR64 ARG61 TRP60 TYR56 ILE52 ALA50 TYR47 GLY38 LEU36
	Furanone C30	− 6.2	SER129 THR115 LEU110 ALA105 PHE101 TYR93 ILE92 TRP88 THR75 PRO74 ASP73 TYR64 TRP60 TYR56
LasR-2UV0	PEA	− 6.5	LYS155 TRP152 LEU151 LEU148 LEU130 SER129 LEU128 ALA127 LEU118 PRO117 THR115 LEU114 PRO85
	OdDHL	− 8.8	SER129 ALA127 GLY126 LEU125 THR115 LEU110 ALA105 PHE101 TYR93 TRP88 CYS79 VAL76 THR75 ASP73 ALA70 TYR64 TRP60 TYR56 ILE52 ALA50 TYR47 LEU40 LEU39 GLY38 LEU36
	Furanone C30	− 5.5	SER129 ALA127 THR115 LEU110 PHE101 TRP88 VAL76 THR75 ASP73 TYR64 ARG61 TRP60 TYR56 LEU36
LasR-6D6O	PEA	− 7.0	SER129 THR115 LEU110 ALA105 PHE102 PHE101 TRP88 VAL76 THR75 ASP73 TYR64 TRP60 TYR56
	OdDHL	− 8.0	SER129 ALA127 GLY126 LEU125 THR115 LEU110 ALA105 PHE101 TYR93 ILE92 TRP88 CYS79 VAL76 THR75 ASP73 TYR64 TRP60 TYR56 ILE52 TYR47 LEU40 LEU39 GLY38 PHE37 LEU36
	Furanone C30	− 6.7	SER129 THR115 LEU110 ALA105 PHE102 PHE101 ILE92 TRP88 THR75 PRO74 ASP73 TRP60 TYR56
LasR-6D6P	PEA	− 7.0	SER129 THR115 LEU110 ALA105 PHE102 PHE101 TRP88 VAL76 THR75 ASP73 TYR64 TRP60 TYR56
	OdDHL	− 8.1	SER129 ALA127 GLY126 THR115 LEU110 ALA105 PHE101 TYR93 ILE92 TRP88 CYS79 VAL76 THR75 PRO74 ASP73 TYR64 ARG61 TRP60 TYR56 TYR47 GLY38 LEU36
	Furanone C30	− 6.6	SER129 THR115 LEU110 TYR56 TRP60 ASP73 PRO74 THR75 TRP88 ILE92 PHE101 PHE102 ALA105
RhIR-4Y15	PEA	− 6.1	SER134 LEU115 ALA110 TRP107 LEU106 PHE100 TRP95 VAL82 ASP80 TYR71 TRP67 TYR63 SER43
	BHL	− 6.6	SER134 LEU115 ALA110 LEU106 PHE100 TRP95 LEU83 VAL82 ASP80 LEU77 TYR71 TRP67 TYR63 SER43
	Furanone C30	− 5.6	SER134 PHE132 TRP95 LEU83 VAL82 ASP80 LEU77 TYR71 TRP67 TYR63 PHE59 CYS45 SER43
RhIR-4Y17	PEA	− 6.8	SER43 CYS45 PHE59 THR61 TYR63 TRP67 VAL68 TYR71 LEU77 ASP80 VAL82 LEU83
	BHL	− 6.8	SER134 LEU115 ALA110 TRP107 LEU106 PHE100 TRP95 LEU83 VAL82 ASP80 TYR71 TRP67 TYR63 CYS45 SER43
	Furanone C30	− 5.9	LEU115 LEU83 ASP80 LEU77 TYR71 VAL68 TRP67 TYR63 THR61 PHE59 CYS45 SER43
CviR-3QP5	PEA	− 6.3	SER155 ILE153 MET135 THR140 ALA130 PHE126 PHE115 TRP111 LEU100 ILE99 ASP97 TYR88 TRP84 TYR80 LEU57
	AHL	− 6.3	SER155 ILE153 THR140 MET135 ALA130 PHE126 PHE115 TRP111 LEU100 ILE99 ASP97 TYR88 TRP84 TYR80 LEU57
	Furanone C30	− 5.3	SER155 ILE153 THR140 MET135 ALA130 PHE126 TRP111 LEU100 ILE99 ASP97 TYR88 TRP84 TYR80 LEU57
CviR′-3QP1	PEA	− 6.7	SER155 ILE153 THR140 MET135 ALA130 PHE126 PHE115 TRP111 ILE99 ASP97 TYR88 TRP84 TYR80 LEU57
	AHL	− 6.6	SER155 ILE153 THR140 MET135 ALA130 PHE126 PHE115 TRP111 LEU100 ILE99 ASP97 LEU57 TYR80 TRP84 TYR88
	Furanone C30	− 5.2	SER155 ILE153 THR140 MET135 ALA130 PHE126 TRP111 LEU100 ILE99 ASP97 TYR88 LEU85 TRP84 TYR80 LEU57

**Figure 6:**
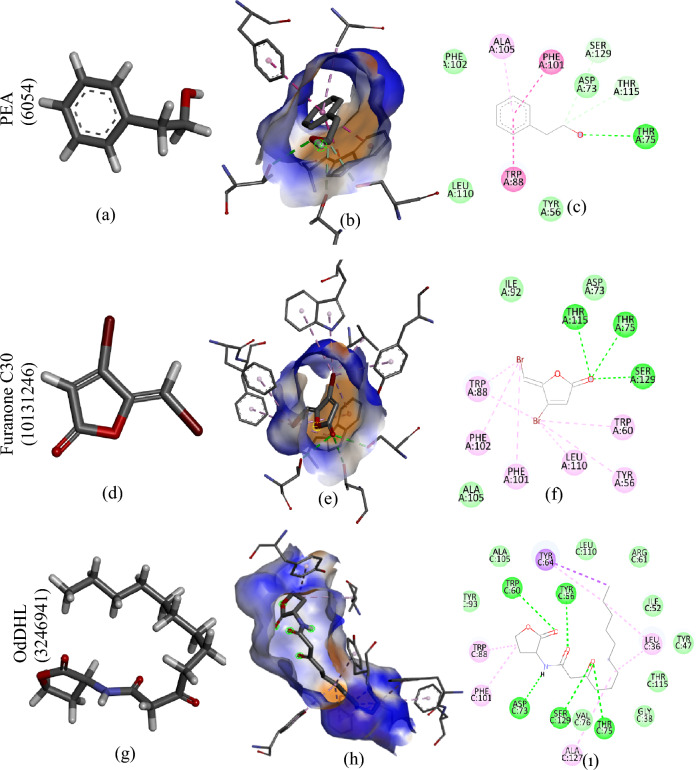
(**a**) 3D structure of PEA; (**b**) 3D structure showing the interaction of LasR-6D6L and PEA (**c**) 2D structure showing the interaction of LasR-6D6L and PEA (**d**) 3D structure of Furanone C30 (**e**) 3D structure showing the interaction of LasR-6D6L and Furanone C30 (**f**) 2D structure showing the interaction of LasR-6D6L and Furanone (**g**) 3D structure of OdDHL (**h**) 3D structure showing the interaction of LasR-6D6L and OdDHL (**ı**) 3D structure showing the interaction of LasR-6D6L and OdDHL.

**Figure 7:**
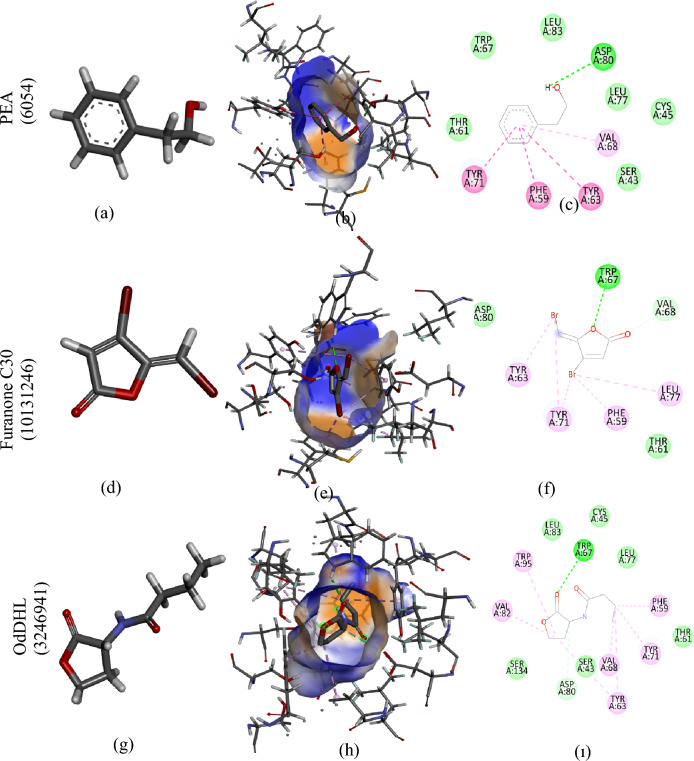
(**a**) 3D structure of PEA; (**b**) 3D structure showing the interaction of RhIR-4Y17 and PEA (**c**) 2D structure showing the interaction of RhIR-4Y17 and PEA (**d**) 3D structure of Furanone C30 (**e**) 3D structure showing the interaction of RhIR-4Y17 and Furanone C30 (**f**) 2D structure showing the interaction of RhIR-4Y17 and Furanone (**g**) 3D structure of BHL (**h**) 3D structure showing the interaction of RhIR-4Y17 and BHL (ı) 3D structure showing the interaction of RhIR-4Y17 and BHL.

In this investigation, molecular docking analysis was conducted to explore the anti-QS mechanism of PEA. We selected five distinct forms of the LasR protein (PDB ID: 2UVO, 6D6L, 6D6A, 6D6O, and 6D6P) and two variants of the RhlR protein (PDB ID: 4EY15, 4EY17) for analysis^67–69^. This analysis aimed to reveal potential mechanisms of QS inhibition by disrupting LasR and RhlR proteins. Detailed information regarding binding affinities and involved amino acid residues is presented in Table 3, supplemented by Figs. 6 and 7.

The structural differences between the LasR and RhlR proteins explain the various binding scores of PEA. Figures 6 and 7 illustrate that PEA interacts well with the LasR-6D6L and RhlR-4Y17 proteins, embedding fully in their active binding pockets. Among all the LasR proteins, PEA exhibited the highest binding affinity to the LasR-6D6L protein (Table 3). To elucidate the interaction between PEA and LasR proteins, we examined its interaction with N-(3-oxododecanoyl)-L-homoserine lactone (OdDHL), the natural ligand of LasR, and the interaction of furanone, a standard inhibitor, with LasR proteins. The binding affinities of PEA, OdDHL, and furanone to the LasR-6D6L protein were calculated as − 7.2, − 7.8, and − 6.6 kcal/mol, respectively. This finding suggests that PEA interacts with the LasR-6D6L protein with higher affinity than furanone. Although PEA's binding affinity to LasR-6D6L was lower than that of OdDHL, it acted competitively.

The binding sites of the PEA ligand to the LasR-6D6L protein (SER129, THR115, LEU110, ALA105, PHE102, PHE101, TRP88, THR75, ASP73, TYR64, TRP60, TYR56) were found to be similar to those of OdDHL and furanone (Table 3). Additionally, previous research on the LasR protein has shown that these same amino acid residues exhibit binding affinity^69^.

The RhlR-4Y15 and RhlR-4Y17 proteins showed similar outcomes (Table 3). Specifically, RhlR-4Y17 demonstrated the highest binding affinity with PEA, significantly surpassing that of furanone-RhlR-4Y17 and equaling the binding affinity of Bhl-RhlR-4Y17. Hydrogen bond interactions are pivotal in stabilizing the ligand significantly. Furthermore, pi-alkyl, van der Waals, and pi-pi interactions also hold substantial importance in ligand binding, enhancing the role of hydrogen bonds. Throughout this investigation, carbon-hydrogen bonds, conventional hydrogen bonds, van der Waals, pi-pi and pi-alkyl interactions emerged as predominant factors.

The findings demonstrate binding competition among PEA, OdDHL, and furanone for identical amino acid residues in all five LasR proteins. Moreover, PEA may bind to LasR proteins better than furanone. This observation also applies to RhlR's 4Y15 and 4Y17 proteins. Notably, the binding affinity of RhlR-4Y17 to its cognate ligand (N-Butanoyl-L-homoserine lactone (BHL)) is equal to its binding affinity to PEA, which is a significant finding. Similarly, the binding affinity of CviR'-3QP1 to its cognate ligand (N-acyl-L-homoserine lactone (AHL)) is lower than its binding affinity to PEA, which is an impressive result.

The PEA ligand interacted with the CviR-3QP5 protein via the amino acid residues SER155, ILE153, THR140, MET135, ALA130, PHE126, PHE115, TRP111, LEU100, ILE99, ASP97, TYR88, TRP84, TYR80, LEU57. This region, where the 3QP5 protein has shown binding affinity, is consistent with data from Bodede et al.^70^. The binding affinity of the PEA ligand to the CviR-3QP5 protein was calculated as − 6.3 kcal/mol, which is equivalent to the binding affinity of the CviR-3QP5 protein to its natural ligand, AHL. Therefore, it can be said that PEA is a good inhibitor for CviR-3QP5. Similar results can be observed for the interaction of PEA with the CviR'-3QP1 protein. The amino acid residues to which the CviR'-3QP1 protein has binding affinity are detailed in Table 3. This region is similar to the active region of the CviR-3QP5 protein and aligns with existing research findings^70,71^.

Hydrogen bond interactions significantly stabilize the ligand. In addition to these, van der Waals, H-bonds, pi-pi, and pi-alkyl interactions are also significant in ligand binding. In this study, conventional hydrogen bonds, carbon-hydrogen bonds, van der Waals, pi-alkyl, and pi-pi interactions seem to dominate. The findings demonstrate binding competition among PEA and OdDHL for identical amino acid residues in all five LasR proteins. In addition, it suggests that PEA may bind to LasR proteins better than furanone. The same is true for RhlR's 4Y15 and 4Y17 proteins. The fact that RhlR-4Y17's binding affinity to the cognate ligand (BHL) is equal to its binding affinity to PEA is a noteworthy finding. Similarly, the binding affinity of CviR'-3QP1 to its natural ligand (AHL) is lower than its binding affinity to PEA. This suggests that the compounds compete for the binding site alongside the signaling molecule.

## Conclusions

This study is the first to investigate the anti-biofilm potential of rose absolute and PEA against *P. aeruginosa* PAO1. Our findings demonstrate that rose absolute and PEA significantly inhibit QS-controlled processes, including EPS production, biofilm formation, swarming motility, and violacein production, without significantly affecting bacterial growth. Molecular docking analyses revealed that PEA interacts with LasR, RhlR, CviR, and CviR' proteins similarly to cognate AHLs, often outperforming the QSI furanone C30. These insights could guide the development of novel antivirulence therapeutic strategies against biofilm-associated infections.

## Data Availability

All data and materials are included within the manuscript.
